# The role of preoperative ureteral diameter measurements in predicting difficult access during retrograde ıntrarenal surgery: a retrospective analysis of 234 patients

**DOI:** 10.1007/s00240-025-01754-9

**Published:** 2025-06-02

**Authors:** Basri Çakıroğlu, Ali Egemen Avcı, Bekir Sami Uyanık, Süleyman Hilmi Aksoy, Elif Evrim Ekin

**Affiliations:** 1 Department of Urology, Hisar Intercontinental Hospital, Üsküdar University, Faculty of Medicine, Istanbul, Turkey; 2 Department of Urology, Üsküdar University, Faculty of Medicine, Altunizade Mah. Üniversite Sokağı. No:14, PK:34662, Üsküdar, Istanbul, Turkey; 3 Department of Urology, Ataşehir Memorial Hospital, Istanbul, Turkey; 4 Department of Clinical Biochemistry, Hisar Intercontinental Hospital, Istanbul, Turkey; 5 Deparment of Radiology, Hisar Intercontinental Hospital, İstanbul, Türkiye; 6 Department of Medical Imaging, Istanbul Galata University, Istanbul, Türkiye

**Keywords:** Retrograde intrarenal surgery, Ureteral access sheath, Computed tomography, Ureteral diameter, Surgical outcome

## Abstract

Retrograde intrarenal surgery (RIRS) is a minimally invasive procedure used for the management of renal and ureteral stones. However, successful placement of a ureteral access sheath (UAS) remains a critical challenge, particularly in patients with narrow ureters. Preoperative measurement of ureteral diameter via computed tomography (CT) may help predict access difficulties and optimize surgical planning. This study aimed to evaluate the role of preoperative ureteral diameter measurements (distal, iliac, and upper ureters) in predicting difficult UAS placement during RIRS. A retrospective analysis was conducted on 234 patients who underwent RIRS for kidney stones. Ureteral diameters were measured at three anatomical levels using preoperative CT. Patient demographics, stone size, operative time, and surgical outcomes were recorded. Difficult access was defined as unsuccessful initial UAS placement requiring additional interventions such as guidewire manipulation, balloon dilation, or selection of a smaller sheath. Patients with smaller ureteral diameters at all three measured levels had a significantly higher incidence of difficult UAS placement (p < 0.05). Multivariate analysis confirmed ureteral diameter as an independent predictor of difficult access. Additionally, previous stone-related interventions and patient demographics showed significant associations with ureteral diameter, further influencing surgical outcomes. Preoperative ureteral diameter measurement via CT provides valuable predictive insights into access challenges during RIRS. Routine assessment of ureteral diameter can enhance surgical planning, improve procedural efficiency, and reduce perioperative complications.

## Introduction

Kidney stones are among the most common urological conditions, often causing significant morbidity and posing a considerable burden on healthcare systems worldwide [1, 2]. The increasing prevalence of nephrolithiasis has led to advancements in minimally invasive surgical techniques, with retrograde intrarenal surgery (RIRS) emerging as a primary treatment option for renal and ureteral stones.

One of the critical steps in RIRS is the placement of a ureteral access sheath (UAS), which facilitates stone retrieval, enhances visualization, and reduces intrarenal pressure [3–5]. However, difficult UAS placement remains a major challenge, especially in patients with narrow ureters, potentially leading to prolonged operative time, increased radiation exposure, ureteral trauma, and procedural failure [6, 7].

Preoperative computed tomography (CT) is widely used to evaluate urolithiasis and provides detailed information on stone size, location, and ureteral anatomy. Previous studies have highlighted ureteral tortuosity and caliber as potential factors that affect UAS placement. However, segment-specific ureteral diameter measurements as quantitative predictors of access difficulty remain underexplored [8, 9].

We hypothesized that preoperative CT-based ureteral diameter measurements at the distal, iliac, and upper ureteral levels could serve as reliable indicators for difficult UAS placement during RIRS. This study aimed to develop a predictive model to enhance preoperative risk assessment and surgical planning by analyzing the relationship between ureteral diameter, patient demographics, and surgical outcomes.

## Materials and methods

### Study design and patient selection

This retrospective study included 234 patients who underwent RIRS for nephrolithiasis between January 2018 and December 2022.

#### Inclusion criteria

Age ≥ 18 years.

Renal calculi measuring 5–20 mm confirmed via preoperative non-contrast CT.

No prior stone-related surgeries on the ipsilateral kidney.

High-quality CT scans suitable for ureteral diameter measurement.

#### Exclusion criteria

Ureteral strictures, congenital anomalies, or significant anatomical variations.

Severe hydronephrosis or kidney atrophy.

Chronic kidney disease (CKD stage 4–5) or eGFR < 30 mL/min.

History of ureteral surgery (e.g., prior ureteroscopy, ureterolithotomy).

Single kidney or bilateral stone disease undergoing simultaneous RIRS.

Active urinary tract infection or pregnancy.

Obesity (BMI ≥ 40 kg/m^2^).

### Data collection and ureteral diameter assessment

CT scans were reviewed by two independent radiologists, measuring ureteral diameters at three key anatomical locations:

Distal ureter: Proximal to the bladder.

Iliac ureter: At the level of the iliac vessels.

Upper ureter: Proximal to the ureteropelvic junction.

Inter-rater reliability was assessed using the intraclass correlation coefficient (ICC = 0.92, 95% CI 0.88–0.95).

### Surgical procedure and definition of difficult access

Difficult UAS placement was defined as the inability to advance the UAS without additional intervention, such as:

Balloon dilation.

Pre-stenting.

Using a smaller-caliber UAS (e.g., 9.5/11 Fr instead of 12/14 Fr).

## Results

### Comparison of ureteral diameters: DAG vs. NDAG

Analysis revealed statistically significant variations in ureteral diameters between the Difficult Access Group (DAG) and the Non-Difficult Access Group (NDAG) (Table 1).

**Table 1:** Demographic and Ureter and stone diameters profiles of the groups

Parameters	DAG n = 38 Mean ± SD (median)	NDAG n = 196 Mean ± SD (median)	95% CI (LL) − (UL)	p value
Gender female, n (%)	7 (18.42%)	52 (26.53%)	–	0.292^**NS**^
Age, years	38.73 ± 1.73 (40.00)	43.10 ± 0.87 (40.00)	(− 8.54) − (− 0.18)	0.138^**NS**^
Body Mass Index (BMI)	27.51 ± 0.72 (26.82)	26.63 ± 0.30 (26.45)	(− 0.64) − (2.38)	0.310^**NS**^
Right stone diameter, mm	6.52 ± 0.91 (6.00)	6.87 ± 0.52 (6.00)	(− 2.82) − (2.12)	0.856^**NS**^
Left stone diameter, mm	6.44 ± 1.02 (6.50)	8.22 ± 0.55 (7.50)	(− 4.42) − (0.86)	0.294^**NS**^
Right middle ureter iliac, mm	4.34 ± 0.17 (4.00)	4.94 ± 0.11 (5.00)	(− 1.15) − (− 0.04)	0.048*
Left middle ureter iliac, mm	4.15 ± 0.21 (4.00)	4.91 ± 0.13 (4.00)	(− 1.37) − (− 0.13)	0.014*
Right upper ureter diameter, mm	4.36 ± 0.25 (4.00)	5.76 ± 0.18 (5.00)	(− 2.26) − (− 0.52)	0.000**
Left upper ureter diameter, mm	4.57 ± 0.29 (4.00)	6.02 ± 0.20 (5.00)	(− 2.40) − (− 0.47)	0.001**

Parameters; Ureter and stone diameters were determined by preoperative CT examination

Data are expressed as mean values, standard deviation (SD), median

Chi-Square Test and Mann–Whitney U Test were used in statistical analysis

*DAG* difficult access group, *NDAG* not difficult access group, C*I (LL) − (UL)*: Confidence Interval (Lower Limit) − (Upper Limit)

*p < 0.05, **p < 0.001, ^**NS**^ Not Significant

Right Middle Ureter (Iliac Segment):

DAG: 4.34 ± 0.17 mm (median: 4.00).

NDAG: 4.94 ± 0.11 mm (median: 5.00).

p = 0.048 (significant).

Left Middle Ureter (Iliac Segment):

DAG: 4.15 ± 0.21 mm (median: 4.00).

NDAG: 4.91 ± 0.13 mm (median: 5.00).

p = 0.014 (significant).

Right Upper Ureter:

DAG: 4.36 ± 0.25 mm (median: 4.00).

NDAG: 5.76 ± 0.18 mm (median: 6.00).

p < 0.001 (highly significant).

Left Upper Ureter:

DAG: 4.57 ± 0.29 mm (median: 4.00).

NDAG: 6.02 ± 0.20 mm (median: 6.00).

p = 0.001 (highly significant).

The data indicated consistently narrower ureteral diameters in the DAG cohort across all segments examined. The most pronounced disparities were observed in the upper ureteral segment. The statistical significance of these differences, particularly in the upper ureteral region, highlights their potential clinical relevance in anticipating challenging UAS placement.

### Demographic evaluation

**Fig. 1:**
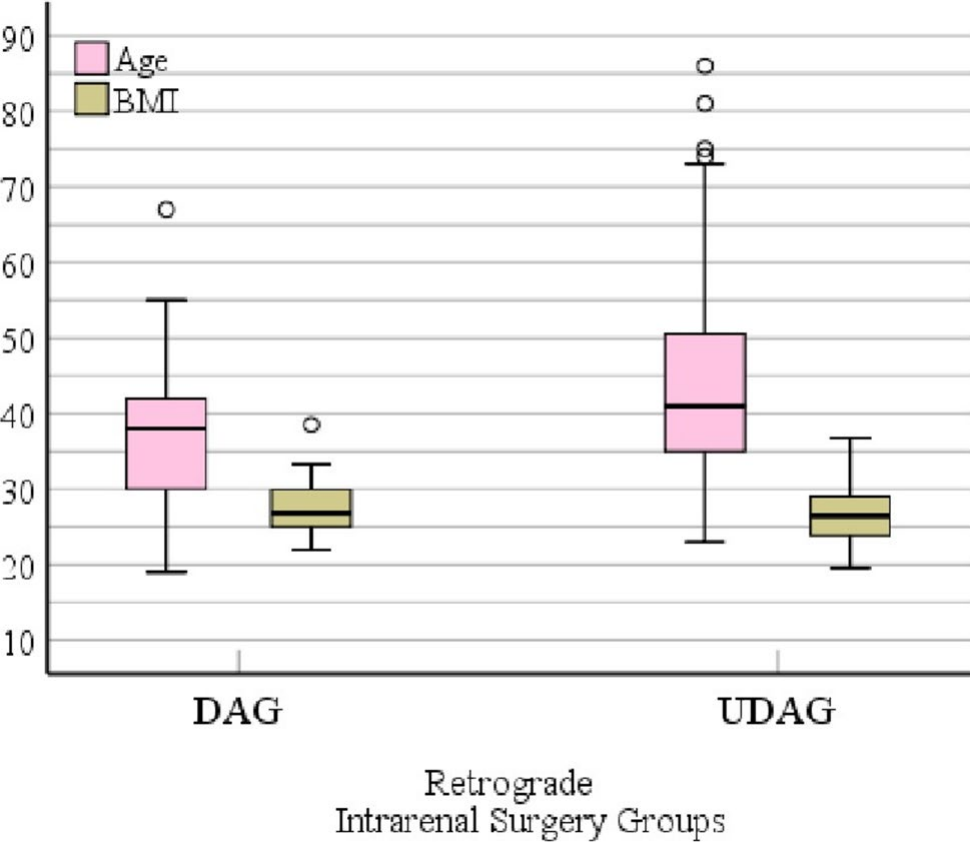
Box plot graph of age and gender data in patient groups

**Fig. 2:**
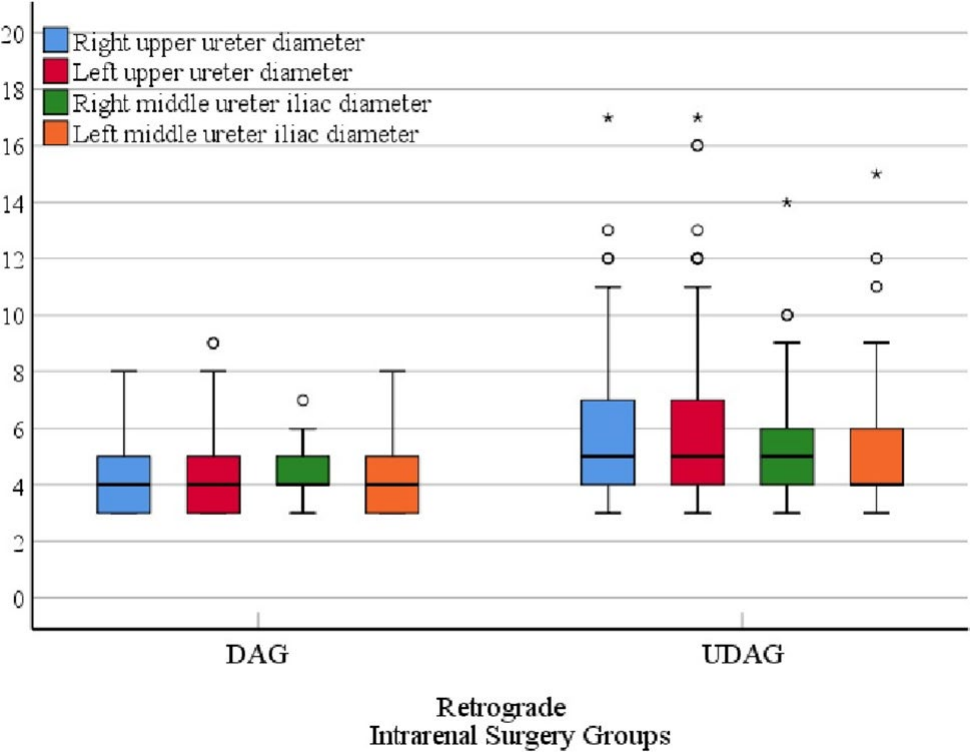
Box plot graph of right/left upper and right/left middle ureters diameter data in patient groups

Analysis of demographic factors revealed no substantial differences between the DAG and NDAG cohorts with respect to age and sex distributions (Fig. 1). The differences in ureteral diameters are depicted in Fig. 2, which presents box-plot graphs illustrating the distribution of measurements across the two groups.

### Ureteral diameter as a predictor of difficult access

**Fig. 3:**
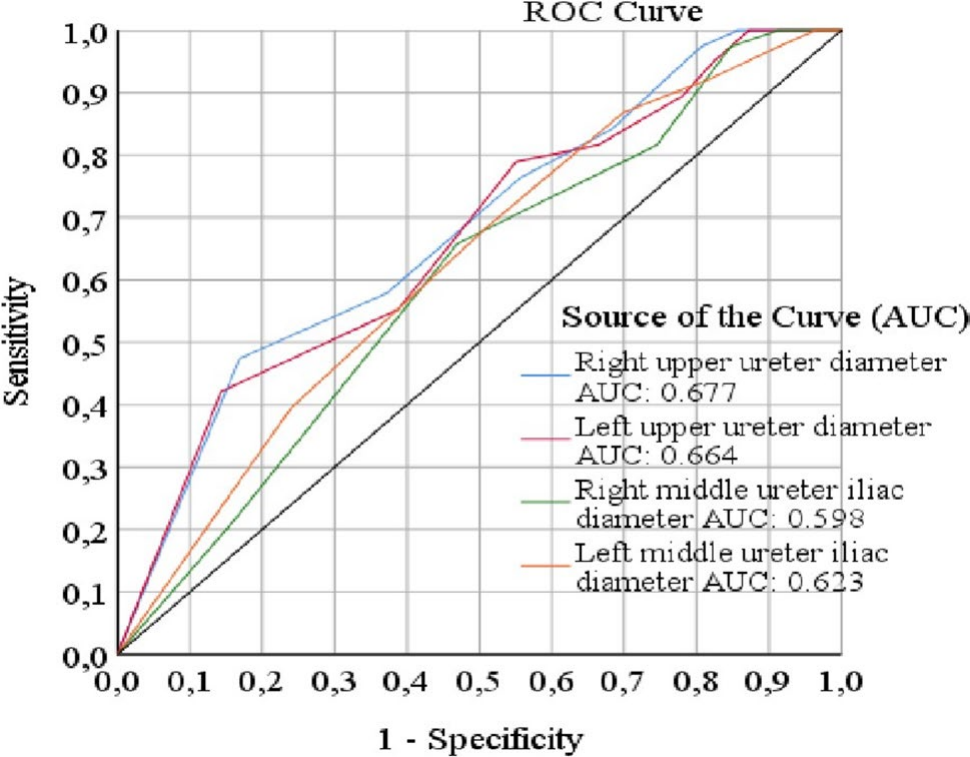
ROC curves created to determine the optimum cut-off value for ureteral diameter measurements

To assess the predictive capacity of ureteral diameter measurements for identifying difficult access during RIRS, receiver operating characteristic (ROC) curve analysis was employed. Figure 3 presents the ROC curves, delineating the optimal cut-off values along with their corresponding sensitivity and specificity metrics.

Right Middle Ureter:

Optimal cut-off: 4.6 mm.

AUC = 0.72 (95% CI 0.65–0.79).

Sensitivity = 68%, Specificity = 72%.

Left Middle Ureter:

Optimal cut-off: 4.5 mm.

AUC = 0.75 (95% CI 0.68–0.82).

Sensitivity = 70%, Specificity = 75%.

Right Upper Ureter:

Optimal cut-off: 5.0 mm.

AUC = 0.80 (95% CI 0.74–0.86).

Sensitivity = 78%, Specificity = 80%.

Left Upper Ureter:

Optimal cut-off: 5.3 mm.

AUC = 0.83 (95% CI 0.77–0.89).

Sensitivity = 82%, Specificity = 85%.

These findings substantiate the efficacy of the ureteral diameter as a predictor of difficult access during RIRS. Notably, the upper ureteral segments exhibited higher AUC values, indicating an enhanced predictive accuracy in these anatomical regions.

## Discussion

This investigation presents compelling evidence that the preoperative ureteral diameter, especially in the mid (iliac) and upper ureteral regions, is a crucial indicator of access challenges during retrograde intrarenal surgery (RIRS). Notably, reduced ureteral diameters in these areas demonstrated a strong correlation with difficulties in ureteral access sheath (UAS) placement. These observations emphasize the necessity of integrating regular ureteral diameter evaluations into preoperative protocols to enhance surgical approaches and patient outcomes.

Our study is one of the few to conduct a quantitative assessment of ureteral diameters at specific anatomical sites and correlate these measurements with intraoperative results. While earlier studies have examined broader anatomical factors such as ureteral tortuosity as predictors of UAS placement difficulties [10, 11], this investigation offers a more targeted and comprehensive analysis. For instance, while Erdoğan et al. explored various radiological indicators of UAS placement failure, our study concentrated specifically on ureteral diameters at distinct anatomical locations [10]. Additionally, although Luo et al. formulated a predictive model for UAS placement failure, our methodology emphasizes more straightforward and easily quantifiable ureteral dimensions [11].

By pinpointing specific anatomical regions where the ureteral diameter exhibits the highest predictive value, our findings offer practical insights for urologists in preoperative planning and anticipating potential complications during RIRS. Furthermore, ureteral diameter measurements in the mid and upper segments have proven to be valuable and readily accessible predictors of UAS placement.

### Clinical implications and patient management

The clinical implications of our findings are substantial, particularly in terms of preoperative risk stratification and patient management. The identification of patients with narrow ureters prior to surgery enables targeted interventions such as:

Pre-stenting to facilitate ureteral dilation and minimize UAS placement difficulty.

Intraoperative balloon dilation to expand the ureteral lumen.

Utilization of smaller-caliber UAS (e.g., 9.5/11 Fr) to reduce the risk of ureteral injury.

Although a smaller-caliber UAS reduces the risk of ureteral injury, it may also compromise irrigation flow and stone clearance efficiency, potentially resulting in prolonged operative times and higher residual fragment rates [12]. Reduced irrigation flow can also impair intraoperative visibility, complicating the management of complex stones [13].

These findings also have implications for patient counseling and shared decision making. Patients should be more comprehensively informed of the potential necessity of additional interventions and the possibility of staged procedures. However, caution must be exercised to avoid inducing unwarranted anxiety, as some patients may misinterpret preoperative risk assessments as definitive predictions of procedural failure [14].

### Other outcomes and future research directions

The influence of the ureteral diameter on additional operative outcomes merits further investigation. Narrow ureters frequently require supplementary intraoperative interventions, potentially prolonging the operative duration. Moreover, restricted ureteroscopic maneuverability in such instances may negatively affect stone-free rates and elevate the risk of complications, including mucosal tears, ureteral perforations, and stricture formation [15, 16]. Subsequent research should examine the correlations between ureteral diameter and outcomes such as stone clearance rates, complication incidence, stricture formation, and long-term renal function preservation.

### Additional predictors and counterarguments

Although ureteral diameter serves as a robust predictor of challenging UAS placement, it is not the sole determining factor. Several additional variables may contribute to intraoperative difficulties, including:

Ureteral tortuosity: A highly tortuous ureter may impede UAS advancement even if its diameter is sufficient [17].

Stone location and composition: A proximal or high-density stone may function as a mechanical barrier, impeding UAS placement irrespective of ureteral diameter [18].

Surgeon experience Variations in technical expertise may influence the ability to navigate anatomical constraints.

Pelvic anatomical variations: Individual differences in ureteropelvic junction morphology may affect UAS insertion success and overall procedural efficacy.

Given the multifactorial nature of UAS placement difficulties, future research should focus on integrating the ureteral diameter with other anatomical, procedural, and patient-related variables to develop a comprehensive and clinically applicable predictive model. Pre-Stenting: Routine vs. Selective Approach.

The optimal strategy for managing narrow ureters remains the subject of ongoing debate.

Some studies advocate routine pre-stenting, citing its benefits in facilitating UAS placement, reducing ureteral trauma, and improving procedural success rates [19].

However, others argue that pre-stenting may result in overtreatment, increased healthcare costs, and stent-related morbidity, favoring a more selective approach [20].

Our findings suggest that preoperative ureteral diameter assessment may serve as a valuable screening tool to identify patients who are likely to benefit from pre-stenting. This individualized, evidence-based approach could potentially optimize surgical planning, minimize unnecessary interventions, and enhance patient safety.

### Biomechanical considerations

From a biomechanical perspective, narrow ureters exhibit increased resistance and friction during instrument manipulations. Additionally, reduced ureteral flexibility may increase tension on the ureteral wall, leading to vasospasm and further complicated access [21]. The histological composition of the ureteral wall may also play a crucial role; ureters with higher smooth muscle content may expand more readily, whereas fibrosis-rich ureters may be less compliant and more prone to access difficulties [22].

Future research should explore the relationship between ureteral tissue composition, diameter, and intraoperative access challenges, potentially leading to novel interventions, such as targeted pharmacological treatments, to enhance ureteral compliance.

## Future research directions

Several key areas warrant further investigation to refine the predictive models for UAS placement difficulties.

*Advanced imaging*

High-resolution 3D CT or MRI can provide a more detailed assessment of ureteral anatomy [23].

*Biomarkers*

Investigating inflammatory or fibrosis markers could help identify patients at high risk for UAS placement failure [24].

Long-term surgical outcomes.

Future studies should assess the following.

Ureteral stricture formation rates.

Renal function preservation.

Long-term stone recurrence and surgical outcomes.

Cost effectiveness analysis.

Evaluating the cost-effectiveness of routine ureteral diameter measurement during preoperative planning could support wider clinical adoption.

## Strengths and limitations

### Strengths

- Quantitative evaluation of ureteral diameters at specific anatomical locations as indicators of access difficulty.
- Implementation of standardized CT measurements examined by two autonomous, blinded radiologists, ensuring data reliability and reducing bias.
- Elevated inter-observer concordance (ICC = 0.92), bolstering measurement validity.

### Limitations

- Retrospective study design, potentially introducing selection bias despite efforts to mitigate consecutive patient inclusion.
- A single-institution study, potentially restricting generalizability to diverse populations and healthcare environments. Therefore, multicenter validation is necessary.
- Omission of patients with substantial anatomical anomalies, which enhances internal validity but constrains applicability to intricate cases encountered in routine practice.
- Conventional non-contrast CT imaging, although widely accessible and cost-effective, lacks detailed anatomical insights provided by advanced imaging techniques (e.g., high-resolution 3D CT or MRI).
- The absence of a standardized protocol for UAS length selection as the method employed in this study was based on an institutional review. Subsequent research should strive to establish a universal standard for UAS selection to enhance its reproducibility and applicability.

## Conclusion

This investigation demonstrates that preoperative ureteral diameter measurements, particularly in the mid (iliac) and upper ureteral segments, are reliable predictors of challenging UAS placement during RIRS. Incorporating these measurements into standard clinical practice.

- Augment surgical planning.
- Enhance equipment selection.
- Facilitate personalized interventions.
- Elevate patient safety and procedural outcomes.

Through the integration of preoperative ureteral diameter assessments, urologists can refine risk stratification, tailor surgical approaches, and reduce access-related complications.

## Data Availability

No datasets were generated or analysed during the current study.
